# Randomized controlled trial of nalfurafine for refractory pruritus in hemodialysis patients

**DOI:** 10.1080/0886022X.2023.2175590

**Published:** 2023-03-01

**Authors:** Ping Zhang, Shilong Xiang, Bicheng Liu, Xiaohui Wang, Xiaoping Yang, Chaoyang Ye, Zunsong Wang, Yanlin Li, Li Zhou, Caili Wang, Hongbo Li, Jian Huang, Ai Peng, Xiaoping Wang, Deguang Wang, Jie Xiao, Wenli Chen, Hong Cheng, Nan Mao, Jianqin Wang, Lin Yang, Jianghua Chen

**Affiliations:** a Kidney Disease Center, the First Affiliated Hospital, College of Medicine, Zhejiang University, Hangzhou, China; b Kidney Disease Center, Key Laboratory of Kidney Disease Prevention and Control Technology, Hangzhou, China; c Kidney Disease Center, National Key Clinical Department of Kidney Diseases, Hangzhou, China; d Institute of Nephrology, Zhejiang University, Hangzhou, China; e Kidney Disease Center, Zhejiang Clinical Research Center of Kidney and Urinary System Disease, Hangzhou, China; f Department of Nephrology, ZhongDa Hospital, Southeast University, Chongqing, China; g Department of Nephrology, Fifth Hospital in Wuhan, Wuhan, China; h Department of Nephrology, The First Affiliated Hospital of Shihezi University School of Medicine, Shihezi, China; i Department of Nephrology, Shuguang Hospital, Shanghai University of Traditional Chinese Medicine, Shanghai, China; j Department of Nephrology, Shandong Province QianFoshan Hospital, Jinan, China; k Department of Nephrology, Zhongshan Traditional Chinese Medicine Hospital, Guangzhou, China; l Department of Nephrology, West China Hospital of Sichuan University, Chengdu, China; m Department of Nephrology, The First Affiliated Hospital of Baotou Medical College, Inner Mongolia University of Science and Technology, Baotou, China; n Department of Nephrology, Wuhan No.1 Hospital, Wuhan, China; o Department of Nephrology, Jinhua Municipal Central Hospital, Jinhua, China; p Department of Nephrology, Shanghai Tenth People’s Hospital, Shanghai, China; q Department of Nephrology, The Central Hospital of Jinan, Jinan, China; r Department of Nephrology, The Second Hospital of Anhui Medical University, Hefei, China; s Department of Nephrology, The First Affiliated Hospital of Guangzhou Medical University, Guangzhou, China; t Department of Nephrology, The Central Hospital of Wuhan, Wuhan, China; u Department of Nephrology, Beijing Anzhen Hospital, Capital Medical University, Beijing, China; v Department of Nephrology, The First Affiliated Hospital of Chengdu Medical College, Chengdu, China; w Department of Nephrology, Lanzhou University Second Hospital, Lanzhou, China; x Department of Nephrology, Yichang Central People’s Hospital, Yichang, China

**Keywords:** Nalfurafine, kappa opioid receptor agonist, hemodialysis, refractory, clinical trial, chronic kidney disease-associated pruritus

## Abstract

**Background:** Chronic kidney disease-associated pruritus (CKD-aP) is very common and sometimes refractory to treatment in hemodialysis patients. In a trial conducted in Japan, nalfurafine, effectively reduced itching of treatment-resistant CKD-aP. Our present bridging study aimed to evaluate the efficacy and safety of nalfurafine in Chinese cohort with refractory CKD-aP.

**Methods:** In this phase III, multicenter bridging study conducted at 22 sites in China, 141 Chinese cases with refractory CKD-aP were randomly (2:2:1) assigned to receive 5 μg, 2.5 μg of nalfurafine or a placebo orally for 14 days in a double-blind manner. The primary end point was the mean decrease in the mean visual analogue scale (VAS) from baseline.

**Results:** A total of 141 patients were included. The primary endpoint analysis based on full analysis set (FAS), the difference of mean VAS decrease between 5 μg nalfurafine and placebo group was 11.37 mm (*p* = .041); the difference of mean VAS decrease between 2.5 μg and placebo group was 8.81 mm, but not statistically significantly different. Both differences were greater than 4.13 mm, which met its predefined success criterion of at least 50% efficacy of the key Japanese clinical trial. The per protocol set (PPS) analysis got similar results. The incidence of adverse drug reactions (ADRs) was 49.1% in 5μg, 38.6% in 2.5 μg and 33.3% in placebo group. The most common ADR was insomnia, seen in 21 of the 114 nalfurafine patients.

**Conclusions:** Oral nalfurafine effectively reduced itching with few significant ADRs in Chinese hemodialysis patients with refractory pruritus.

## Introduction

Chronic kidney disease-associated pruritus (CKD-aP) is a very frequent and frustrating problem for both patients and clinicians. The incidence of CKD-aP varied between 40.0% and 72.5% of patients undergoing hemodialysis in the literature [1–5], and about 20% to 40% of patients reported moderate to severe pruritus [3]. Refractory pruritus not only significantly impacted patients’ quality of life, but also resulted in poor prognosis. Intense and generalized systemic itching were associated with poor sleep quality, depression, reduced quality of life, increased risk of infection and higher mortality risk [3,6–10]. Unfortunately, current treatments with various conventional drugs are often not satisfactorily effective and therapeutic options are sparse for severe CKD-aP [11–16]. The current literature supports the use of gabapentinoids as an effective treatment in CKD-aP [15], but the significant adverse risks must be informed and monitored carefully [17]. New drugs are greatly expected to be developed.

It has been shown that an imbalance of endogenous opioid system may be one contributor to the itch of CKD-aP, and the μ-opioid system is itch-inducing, whereas the κ-opioid system is itch-suppressive [11,18,19]. Nalfurafine hydrochloride was a selective κ-opioid receptor agonist, which was first invented by Toray in 1992. A randomized comparative study demonstrated that intravenously administered nalfurafine was effective in relieving the itch compared to placebo for CKD-aP undergoing routine hemodialysis [20]. A follow-up randomized, prospective, placebo-controlled phase III study showed that orally taken nalfurafine effectively reduced refractory itch, with few adverse drug reactions (ADRs) [19]. No evidence of abuse liability was indicated in a long-term study [21]. Post-marketing surveillance study of oral nalfurafine continued to be safe and effective for intractable pruritus in hemodialysis patients in real-world clinical settings [22].

A limitation of the above clinical trials was that the registered patient populations had the same background. In some ways, there is ethnic difference between Chinese and Japanese populations, which may alter the drug’s safety, efficacy, or dose response. Therefore, it is important to know whether the result from the previous phase III trial conducted in Japan [19] can be extrapolated to a Chinese population. As described in the International Conference of Harmonization (ICH) E5 guidelines [23,24], the role of bridging study is to allow safe drug approval in ethnically different countries without duplication of research efforts. In the present report, we designed and carried out a prospective, Phase III, randomized, double-blind, placebo-controlled, multicenter, bridging study to evaluate the efficacy and safety of nalfurafine hydrochloride in Chinese hemodialysis patients with refractory CKD-aP.

## Methods

### Patients

This study enrolled patients on hemodialysis who had been on stable dialysis for 3 months or more, received regular hemodialysis 3 times a week. All patients had ‘existing treatment-resistant’ pruritus, also known as refractory pruritus [19,25,26], defined as pruritus responding inadequately to systemic therapy (with oral or injectable prescription gabapentin, or antihistamines or other anti-allergic drugs, such as glucocorticoids, sodium thiosulfate) at least 2 consecutive weeks and topical treatment (ointments or moisturizers prescribed by physicians), during the 1-year period before signing the informed consent. The study protocol is in accord with the Helsinki declaration. The study protocol was approved by an internal review board at each research center that participated in the study. The clinical trial was registered on clinical trial (https://clinicaltrials.gov/show/NCT04728984, grant number NCT04728984) and China drug trials (http://www.chinadrugtrials.org.cn, grant number: CTR20201271). All subjects provided signed informed consent before participating in the trial. A list of inclusion and exclusion criteria is provided in Table S1 in the Supplementary Appendix.

**Table 1.** Characteristic of patients at baseline (FAS).

	Characteristic	5μg *N* = 57	2.5μg *N* = 57	Placebo *N* = 27	Total *N* = 141
Gender[n(%)]	Male	40 (70.2)	46 (80.7)	21 (77.8)	107 (75.9)
	Female	17 (29.8)	11 (19.3)	6 (22.2)	34 (24.1)
Age (years)	Mean (SD)	57.2 (14.33)	53.7 (12.35)	56.6 (14.51)	55.7 (13.60)
Race[n(%)]	Han	57 (100.0)	57 (100.0)	25 (92.6)	139 (98.6)
	Others	0 (0)	0 (0)	2 (7.4)	2 (1.4)
Primary disease, n(%)	Glomerulonephritis	26 (45.6)	23 (40.4)	10 (37.0)	59 (41.8)
	Diabetic nephropathy	10 (17.5)	12 (21.1)	10 (37.0)	32 (22.7)
	Other etiologies	21 (36.8)	22 (38.6)	7 (25.9)	50 (35.5)
Mean VAS value (mm) in the pre-observation period (latter seven days)	Mean (SD)	83.0 (11.0)	82.0 (9.5)	82.3 (8.5)	
Duration of pruritus (months)	Mean (SD)	27.28 (32.04)	33.77 (36.95)	35.59 (43.30)	31.50 (36.27)
Undergo stable dialysis	Yes (%)	57 (100.0)	57 (100.0)	27 (100.0)	141 (100.0)
Duration of dialysis (months)	Mean (SD)	66.90 (45.41)	71.30 (44.51)	62.40 (43.54)	67.80 (44.49)
Frequency of weekly dialysis (times/week)	1	0	0	0	0
	2	0	0	0	0
	3	57 (100.0)	57 (100.0)	27 (100.0)	141 (100.0)
Laboratory findings at baseline					
Hemoglobin (g/L)	Mean (SD)	113.8 (16.26)	109.7 (17.75)	114.1 (16.16)	
Eosinophil count(×10^9^/L)	Mean (SD)	0.33 (0.30)	0.29 (0.27)	0.29 (0.23)	
Albumin (g/L)	Mean (SD)	41.00 (4.04)	40.30 (3.64)	41.14 (5.40)	
Total bilirubin (umol/L)	Mean (SD)	7.91 (6.93)	7.49 (3.10)	7.71 (3.40)	
Glutamate pyruvate transaminase (IU/L)	Mean (SD)	14.34 (9.01)	12.70 (9.22)	14.32 (12.58)	
Serum creatinine (mg/dL )	Mean (SD)	11.19 (2.80)	11.55 (2.97)	10.69 (3.73)	
Serum urea nitrogen(mmol/L)	Mean (SD)	26.58 (7.13)	26.53 (7.94)	27.25 (10.25)	
Calcium (mmol/L)	Mean (SD)	2.27 (0.25)	2.26 (0.24)	2.28 (0.27)	
Phosphorus (mmol/L)	Mean (SD)	2.24 (0.77)	2.10 (0.78)	2.24 (0.82)	
Testosterone (ng/ml)	Mean (SD)	2.40 (1.74)	2.73 (1.57)	2.62 (1.64)	
Prolactin (ng/ml)	Mean (SD)	33.66 (27.52)	33.80 (38.60)	44.52 (44.50)	
Thyroid stimulating hormone (mIU/L)	Mean (SD)	1.85 (1.40)	2.11 (1.43)	3.34 (3.92)	
Drugs in use[n(%)]	Calcium channel blockers	38 (66.7)	46 (80.7)	18 (66.7)	102 (72.3)
	β-blockers	36 (63.2)	41 (71.9)	15 (55.6)	92 (65.2)
	RAAS inhibitors	28 (49.1)	32 (56.1)	13 (48.1)	73 (51.8)
	Diuretics	6 (10.5)	2 (3.5)	1 (3.7)	9 (6.4)
	Topical treatment	45 (78.9)	43 (75.4)	23 (85.2)	111 (78.7)
	Antihistamines drugs	10 (17.5)	17 (29.8)	5 (18.5)	32 (22.7)
	Anti-allergic drugs	3 (5.3)	0 (0)	1 (3.7)	4 (2.8)
	Gabepentin	3 (5.3)	1 (1.8)	0 (0)	4 (2.8)

Abbreviations: FAS: full analysis set; SD: standard deviation; RAAS: renin-angiotensin-aldosterone system.

### Study design

This study was designed as a randomized, double-blind, placebo-controlled, multicenter bridging study in which three groups were randomly (2:2:1) treated with nalfurafine hydrochloride (5 or 2.5 μg) (Toray Industries, Inc.) or a placebo (Toray Industries, Inc.) orally after dinner once daily, as shown in detail in the Randomization Schemes in the Supplementary Appendix. The study was composed of a 14-day pre-observation period, a 14-day drug treatment period, and an 8-day post-observation period. Throughout the study, conventional antipruritic drugs were continuously administered at the same dosage and administration schedule as used at baseline. The study design and flow chart are shown in Table S2 in the Supplementary Appendix.

**Table 2.** The summary of primary endpoint.

	Groups	LS means	95%CI	*p* Value
FAS, LOCF				
Step one： 5μg nalfurafine VS placebo	5μg Nalfurafine	31.21	(25.05, 37.37)	
	Placebo	19.84	(10.89, 28.80)	
	5μg Nalfurafine group - placebo	11.37	(0.50, 22.24)	.041
Step two： 2.5 μg nalfurafine VS placebo	2.5 μg Nalfurafine	28.40	(22.25, 34.54)	
	Placebo	19.59	(10.66, 28.52)	
	2.5μg Nalfurafine group - placebo	8.81	(−2.03, 19.65)	.110
PPS				
Step one： 5μg nalfurafine VS placebo	5μg Nalfurafine	32.10	(25.84, 38.37)	
	Placebo	19.82	(11.04, 28.61)	
	5μg Nalfurafine-placebo	12.28	(1.48, 23.07)	.026
Step two： 2.5 μg nalfurafine VS placebo	2.5 μg Nalfurafine	29.03	(22.51, 35.54)	
	Placebo	19.48	(10.43, 28.52)	
	2.5 μg Nalfurafine-placebo	9.55	(−1.60, 20.70)	.092

Abbreviations: FAS: full analysis set; LOCF: last-observation-carried-forward; PPS: per protocol set; LS means: least-squares means; CI: confidence interval.

### Itch measurement

We evaluated the itch severity in 2 ways. First, itch severity was measured with visual analogue scale (VAS), which is one of the most frequently used methods for pruritus assessment [27,28]. The VAS consisted of a 100 mm horizontal line measured in millimeters with no scale markings, in which the left end of the line (0 mm) represented no itching and the right end (100 mm) the worst itching imaginable [29]. In the absence of physicians or other staffs, each patient/candidate was instructed to mark the subjective sensation of their itch disturbance on the scale to record the highest severity experienced during the previous 12 h twice daily (once in the morning and once in the evening) throughout the study period (for 36 days). The VAS is seen in Figure S1 in the Supplementary Appendix.

Second, the intensities of daytime and nighttime itching were measured with the Xie-kawashima itch scale [30]. The mean larger Xie-kawashima itch scale of each day both in the morning and evening, were calculated for the last seven days of the pre-observation period, and latter seven days of the treatment period. In this assessment, itch severity was scored from 0 (absent) to 4 (intense) (Table S3 in the Supplementary Appendix).

**Table 3.** The summary change of mean larger Xie-kawashima itch scale between latter seven-day pre-observation and latter seven-day treatment period.

	Groups	LS means	95%CI	*p* Value
5μg Nalfurafine VS placebo	5μg Nalfurafine	1.37	(1.12, 1.61)	
	Placebo	1.13	(0.77, 1.48)	
	5μg Nalfurafine-placebo	0.24	(−0.19, 0.67)	.271
2.5 μg Nalfurafine VS placebo	2.5μg Nalfurafine	1.23	(0.99, 1.47)	
	Placebo	1.14	(0.80, 1.49)	
	2.5 μg Nalfurafine-placebo	0.09	(−0.33, 0.51)	.673

Abbreviations: LS means: least-squares means; CI: confidence interval.

### Endpoints

#### Primary endpoint

##### Change in VAS value

Evaluating the VAS values both in the morning and evening, the daily larger VAS value was defined as the larger one of each day. And the mean larger VAS values of each day were calculated for the last seven days of the pre-observation period, and latter seven days of the treatment period. Using the mean larger VAS value for the last seven days of the pre-observation period as a baseline, the decrease in the mean VAS value from the baseline during each subsequent period was assessed as the change in the VAS value. The primary endpoint was defined as the change from the mean larger VAS value of the last seven days of the pre-observation period and the mean larger VAS value of the latter seven days of the treatment period.

#### Secondary endpoint

1. **Evaluate the Xie-kawashima itch scale**
  Both in the morning and evening, the mean larger Xie-kawashima itch scale of each day were calculated for the last seven days of the pre-observation period, and latter seven days of the treatment period. The mean larger Xie-kawashima itch scale for the last seven days of the pre-observation period was used as a baseline. The secondary endpoint was defined as the change of the mean larger Xie-kawashima itch scale of the latter seven days of the treatment period compared the baseline level.
2. **Improvement of sleep disorder caused by pruritus**
  The mean Xie-kawashima itch scale of each evening were calculated for the last seven days of the pre-observation period, and latter seven days of the treatment period. Improvement of sleep disorder caused by pruritus was evaluated by the change of the mean Xie-kawashima itch scale in the evening.
3. **Improvement degree of VAS value**
  Using all the VAS values both in the morning and evening, the mean VAS values were calculated for the last seven days of the pre-observation period, the latter seven days of the treatment period. Using the mean VAS value for the last seven days of the pre-observation period as a baseline. The improvement degree of VAS value was assessed as the decrease in the mean VAS value from the baseline. And its improvement was defined as follows [31]:
  **Very effective**: The mean VAS values for the last seven days of the treatment period is less than 20 mm or it decreased more than 40 mm from the baseline.
  **Effective**: The decrease of mean VAS values for the last seven days from the baseline is between 20 mm and 40 mm, which cannot be judged as very effective.
  **Invalid**: Other situations that cannot be judged as very effective or effective.

All of the above secondary endpoints analysis were based on the full analysis set (FAS).

### Statistical analysis

The predefined success criteria of our bridging trial was the difference of VAS decreases greater than 4.13 mm, retained at least 50% of the efficacy (8.26 mm) of the key clinical trial in Japan. To meet the primary purpose of this bridging trial, by assuming an expected difference of 8.26 mm in the change in VAS values, with the standard deviation of 20mm and a statistical power of 80%, the sample size of 125 Chinese patients were needed in the FAS: among them, 50 cases were in the 5 μg dose group of nalfurafine, 50 cases were in the 2.5 μg group, and 25 cases were in the placebo group.

The FAS was the main population for efficacy analysis. It included all participants who were randomized, received at least one dose of the study drug, and evaluated of post-treatment efficacy at least once. The per protocol set (PPS) was a subset of the full analysis set and included all subjects who met the inclusion/exclusion criteria specified in the protocol, had good compliance, and had no major protocol deviations during the trial. The PPS set was a secondary population for efficacy analysis. The safety analysis set (SS) included all randomized participants who received at least one dose of the study drug, and obtained safety assessment at least once.

In the primary endpoint analysis, the missing data were imputed with the use of the last-observation-carried-forward (LOCF) method. And in the analysis of secondary endpoint the missing data were not imputed. It is a bridging clinical study. The primary endpoint goal is that the difference of the change in average VAS between the study group and the placebo control group is no less than 4.13 mm (at least should get the 50% of the curative effect in the key phase III clinical trial in Japan). The results of bridging study will be tested by the fixed sequence test method, the 5 μg nalfurafine to placebo groups and the 2.5 μg nalfurafine to placebo groups will be tested respectively. The analysis of covariance (ANCOVA) method was used for comparison between groups. The average value of the larger value of VAS measured at the time of getting up and going to bed in the latter seven-day pre-observation period was used as the covariate. The two-sided 95% confidence interval (CI) was determined for the intergroup difference in the mean VAS values. Statistical analyses were performed using SAS version 9.4.

## Results

### Patients

**Figure 1.**
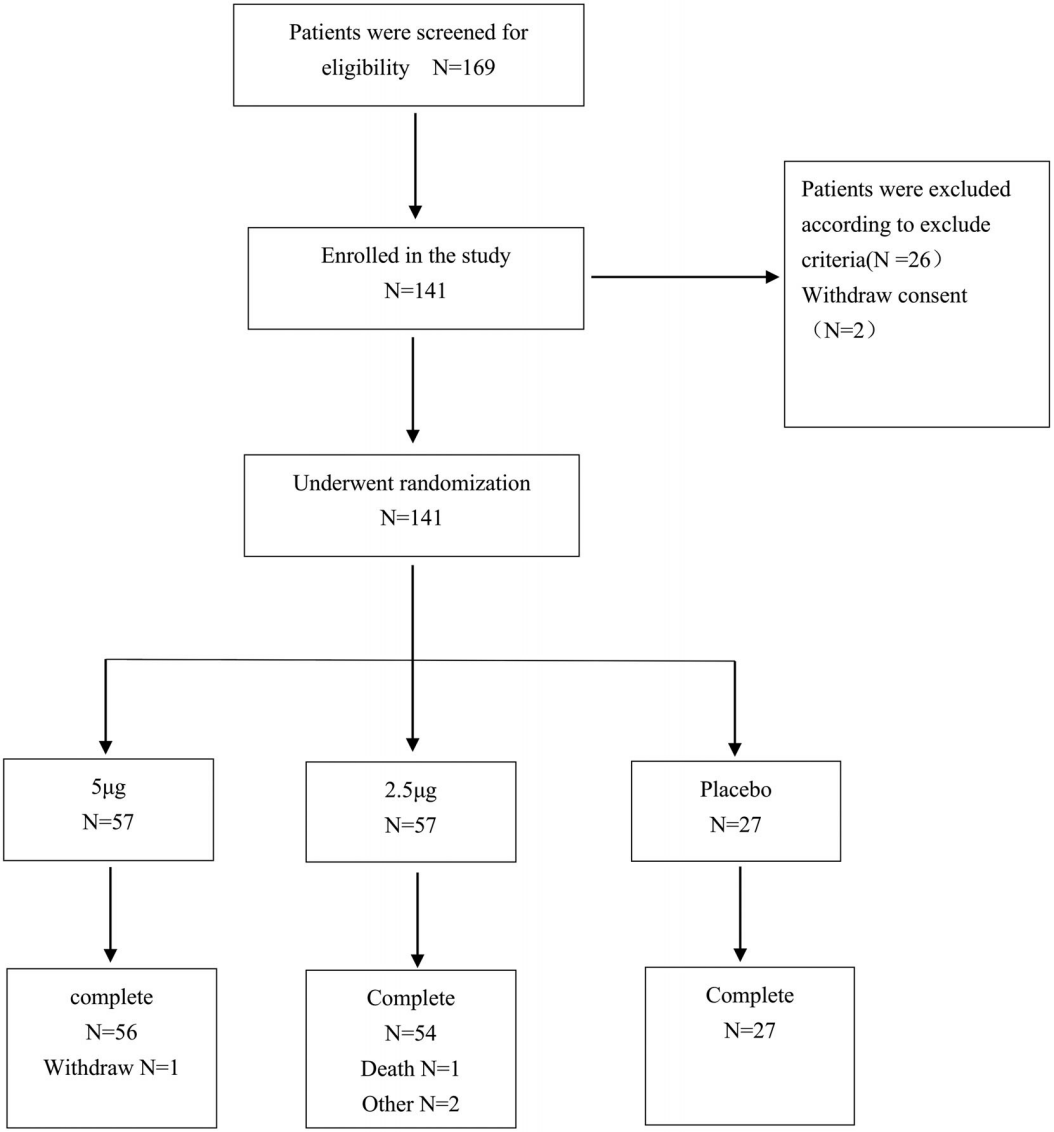
Flow diagram of the progress through the randomized trial.

The study was carried out at 22 centers throughout China, and 169 hemodialysis patients with severe refractory pruritus were assessed for eligibility (Figure 1). A total of 141 subjects were randomly included and all of them were analyzed in the FAS and SS, and a total of 132 subjects were included in the PPS. And the details of patients’ characteristics are shown in Table 1.

### Efficacy

#### Primary endpoint

Based on FAS, in the primary endpoint analysis, the missing data will be imputed by carried forward by the last observation value respectively. In the intergroup comparison in step one, the least square mean of VAS change was 31.21 mm in the 5 μg nalfurafine group, and 19.84 mm in the placebo group. The difference of 11.37 mm between two groups is greater than 4.13 mm, the original hypothesis 50% effect of the Japanese phase III clinical trial, with a significant difference (*p* = .041).

And then in step two, the least square mean of VAS change was 28.40 mm in the 2.5 μg nalfurafine group, and 19.59 mm the placebo group. The difference of mean VAS decrease between two groups was 8.81 mm, though no significant difference (*p* = .110). So, based on FAS, the effect of 5 μg and 2.5 μg groups were consistent with the result of phase III study in Japan and got the bridge study goal.

So as to PPS, the least square mean of VAS change in the 5 μg nalfurafine group was 32.1 mm, and in the placebo group was 19.82 mm. The difference of 12.28 mm between two groups was statistically significant (*p* = .026). When compared between the 2.5 μg nalfurafine group and placebo group, the least square mean of VAS change was 29.03 mm and 19.48 mm respectively, and the difference between two groups was 9.55 mm (*p* = .092). Both the differences were greater than 4.13 mm, the original hypothesis 50% effect of the Japanese phase III clinical trial. Similar to FAS, the results achieved the bridge study goal. We believed that this trial had obtained a positive result and successfully achieved the purpose of the bridging trial. The summary of primary endpoint is shown in Table 2.

#### The secondary endpoints

**Figure 2.**
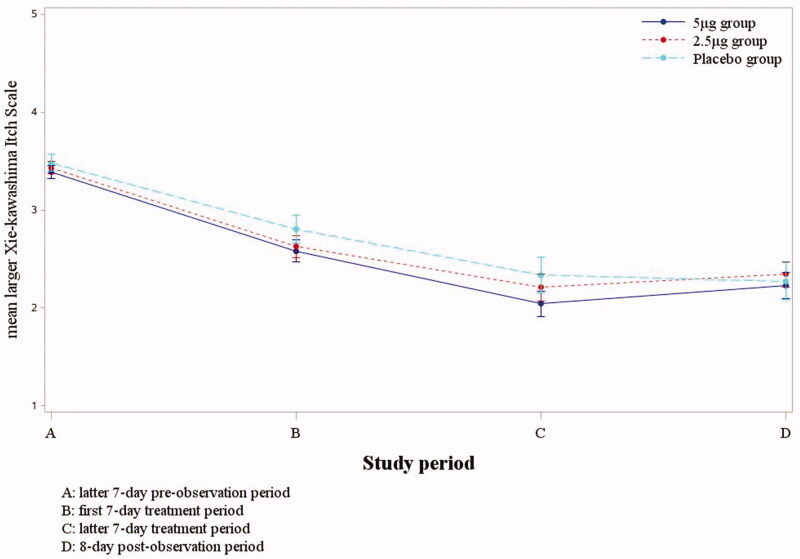
The trend of mean larger Shiratori’s severity score during the study period.

**Figure 3.**
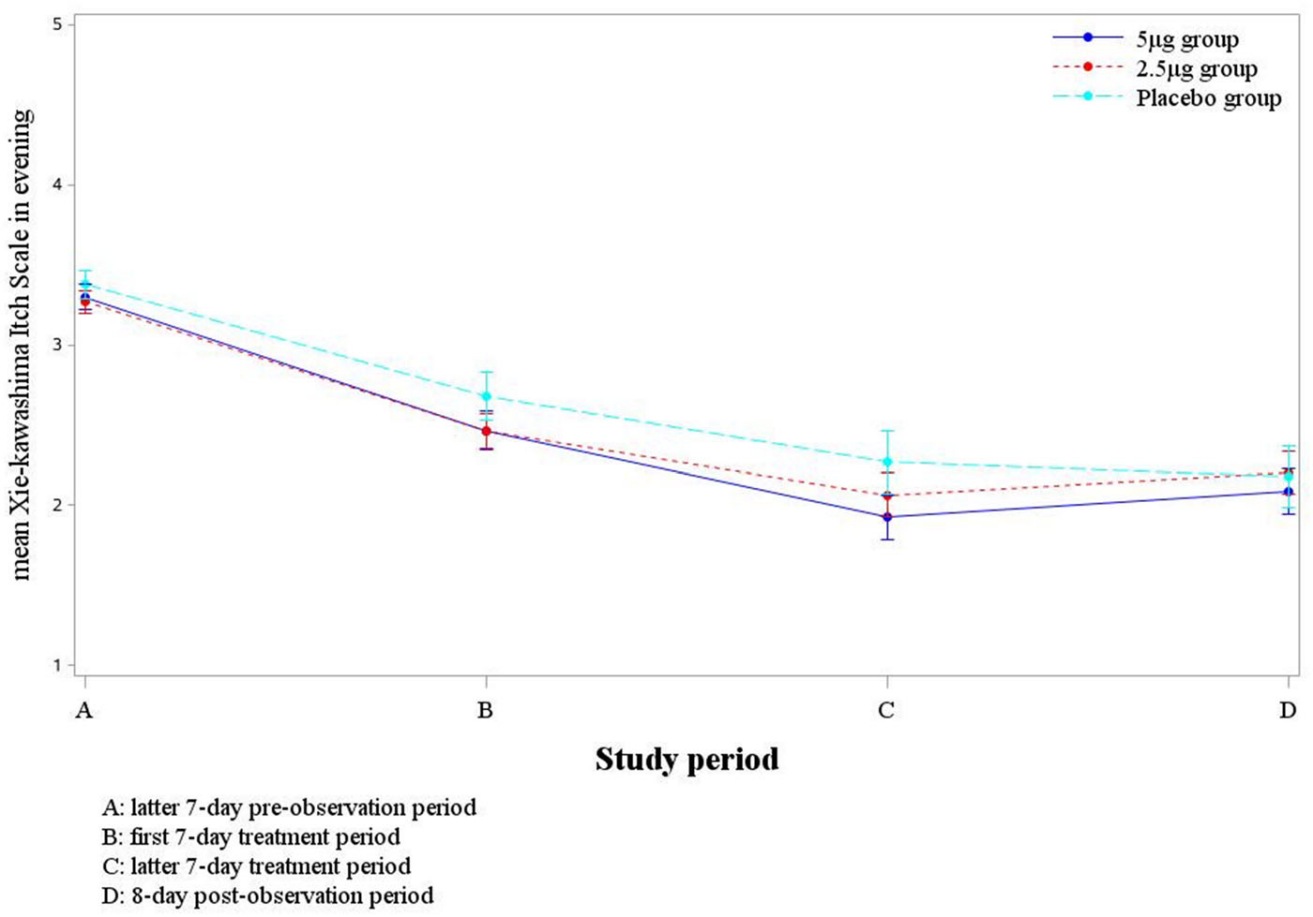
The trend of mean Shiratori’s severity score in evening during the study period.

1. **Change of mean larger Xie-kawashima itch scale**
  The change of Xie-kawashima itch scale of the latter seven-day pre-observation and latter seven-day treatment period were as follows: when compared between the 5 μg nalfurafine group and placebo group, the least squares means of the 5 μg nafurafine group was 1.37, and the placebo group was 1.13, with a difference of 0.24 (*p* = .271). When compared between the 2.5 μg nalfurafine group and placebo group, the least squares mean was 1.23 and 1.14 respectively, with a difference of 0.09 (*p* = .673) (as shown in Table 3).
  The results showed that the overall trend of the three groups in Xie-kawashima itch scale. The daily larger pruritus score of the test group based on Xie-kawashima itch scale evaluation method showed a downward trend after administration, reaching the lowest in latter seven-day treatment period, and the decline range was greater than that of the placebo group, and the 5 μg nalfurafine group seemed to be greater than the 2.5 μg nalfurafine group, although no statistical difference. Both research groups rose again after treatment period, while in the placebo group, it still showed a downward trend at eight-day post-observation period (as shown in Figure 2).
2. **Improvement of sleep disorder caused by pruritus**
  The change of Xie-kawashima itch scale of each evening between the latter seven-day pre-observation and latter seven-day treatment period as follows: when compared between the 5 μg nalfurafine group and placebo group, the least squares means of the 5 μg nafurafine group was 1.40, the placebo group was 1.09, with a difference of 0.30 (*p* = .192). When compared between the 2.5 μg nalfurafine group and placebo group, the least squares mean of 2.5 μg nafurafine group was 1.21, the placebo group was 1.10, with a difference of 0.11 (*p* = .615) (as shown in Table 4).
  Figure 3 shows that the change of mean Xie-kawashima itch scale of each evening, the treatment groups were greater than placebo group, and the 5 μg nalfurafine group seemed to be greater than the 2.5 μg nalfurafine group, although no significant difference.
3. **Improvement degree of VAS value**
  Based on FAS analysis, the very effective rate in the 5 μg nalfurafine group, 2.5 μg nalfurafine group and placebo group was 38.6% (22 cases), 28.1% (16 cases), 14.8% (4 cases) respectively, and the total effective rate was 59.6%, 52.6%, 44.4% respectively. The total effective rate of both nalfurafine treatment groups seemed to be greater than the placebo group, although no significant difference (*p* > .05) (as shown in Table 5).

**Table 4.** The summary change of mean Xie-kawashima itch scale in evening between latter seven-day pre-observation and latter seven-day treatment period.

	Groups	LS means	95%CI	*p* Value
5μg Nalfurafine VS placebo	5μg Nalfurafine	1.40	(1.14, 1.66)	
	Placebo	1.09	(0.72, 1.47)	
	5μg Nalfurafine-placebo	0.30	(−0.15, 0.76)	.192
2.5 μg Nalfurafine VS placebo	2.5 μg Nalfurafine	1.21	(0.97, 1.46)	
	Placebo	1.10	(0.75, 1.45)	
	2.5 μg Nalfurafine-placebo	0.11	(−0.32, 0.54)	.615

Abbreviations: LS means: least-squares means; CI: confidence interval.

**Table 5.** The summary of improvement of VAS value and effect.

Groups	5μg Nalfurafine	2.5μg Nalfurafine	Placebo
Number of patients	56	56	27
Invalid	22 (38.6%)	26 (45.6%)	15 (55.6%)
Effective	12 (21.1%)	14 (24.6%)	8 (29.6%)
Very effective	22 (38.6%)	16 (28.1%)	4 (14.8%)
Effective rate(95%CI)	59.6 (45.82,72.44)	52.6 (38.97,66.02)	44.4 (25.48,64.67)
*p* Value (vs placebo)	.191	.483	

Abbreviation: CI: confidence interval.

#### Safety

During the treatment period of study, there were 136 adverse events (AEs) occurred in 44 subjects (77.2%) in the 5 μg nalfurafine group, 112 AEs in 45 subjects (78.9%) in the 2.5 μg nalfurafine group, and 66 AEs in 23 subjects (85.2%) in the placebo group. There are total of four drop-outs during the trial: one patient due to withdrawal of informed consent in the 5 μg group, three patients in the 2.5 μg group, of which one due to death, one due to serious adverse event (SAE), one due to AE. The percentage of patients who completed the study was 97.16%.

AEs were assessed according to Common Terminology Criteria for Adverse Events version 5.0 (CTCAE v5.0). The AE severity in this study were mainly grade 1 and grade 2. Of treatment-emergent adverse events severer than grade 3, there were 3 cases in 3 patients in the 5 μg nalfurafine group, 13 cases in 6 patients in the 2.5 μg nalfurafine group, and 4 cases in 3 patients in the placebo control group. A total of 6 subjects had serious adverse events (SAEs) during the treatment period (including 1 case of dead of cholangitis), and all SAEs were not to be related to the study drug. There were no adverse reactions leading to withdrawal from the study in each group.

The most frequent ADR in this study was insomnia [32], which was occurred in 13 cases (22.8%) of the 5 μg nalfurafine group, in 8 cases (14%) of the 2.5 μg nalfurafine group and in 4 cases (14.8%) of the placebo group respectively. In addition to insomnia, the incidence of ADRs greater than 5% included: blood prolactin increased (21.1%), irritability (5.3%) and nausea (7.0%) in the 5 μg nalfurafine group; blood prolactin increased (8.8%) in the 2.5 μg nalfurafine group; blood prolactin increased (7.4%) and thirsty (7.4%) in the placebo group. In terms of laboratory examination, about the endocrinology examination items, the testosterone, thyroid stimulating hormone and thyroxine of both treatment groups tended to decrease during administration, while blood prolactin of the 5 μg nalfurafine group tended to increase, afterward basically back to the baseline level on the day of follow-up. In the previous Japanese phase III trial [19], transient change of prolactin, thyroid stimulating hormone and testosterone were found. Therefore, we paid close attention to the changes of these hormones. In addition, no abnormalities requiring special attention were found in other laboratory examinations, vital signs, physical examinations and electrocardiograms (as shown in Table 6).

**Table 6.** The summary of adverse events.

Item	5 μg group (*n* = 57)	2.5 μg group (*n* = 57)	Placebo group (*n* = 27)
	No. of patients (%)		
Adverse events^a^			
Insomnia	13 (22.8)	8 (14.0)	4 (14.8)
Irritability	3 (5.3)	2 (3.5)	0
Nausea	5 (8.8)	0	1 (3.7)
Thirsty	1 (1.8)	2 (3.5)	2 (7.4)
Palpitation	1 (1.8)	0	2 (7.4)
Muscle spasms	4 (7.0)	2 (3.5)	1 (3.7)
Urinary tract infection	0	3 (5.3)	0
Upper respiratory tract infection	0	3 (5.3)	0
Blood prolactin increased	12 (21.1)	5 (8.8)	2 (7.4)
Blood testosterone decreased	2 (3.5)	1 (1.8)	2 (7.4)
Hypothyroidism	0	0	2 (7.4)
Leukocytosis	4 (7.0)	3 (5.3)	0
Hyperlipidemia	5 (8.8)	2 (3.5)	5 (18.5)
Hyperkalemia	1 (1.8)	4 (7.0)	3 (11.1)
Hypercalcemia	0	0	2 (7.4)
Adverse drug reactions^a^			
Insomnia	13 (22.8)	8 (14.0)	4 (14.8)
Irritability	3 (5.3)	2 (3.5)	0
Nausea	4 (7.0)	0	1 (3.7)
Thirsty	1 (1.8)	2 (3.5)	2 (7.4)
Blood prolactin increased	12 (21.1)	5 (8.8)	2 (7.4)
Serious adverse events			
Rhabdomyolysis	1 (1.8)	0	0
Chronic obstructive pulmonary disease	1 (1.8)	0	0
Polycystic kidney hemorrhage	0	1 (1.8)	0
Traffic accident trauma	0	1 (1.8)	0
Quadriceps tendinousrupture	0	1 (1.8)	0
Cholangitis	0	1 (1.8)	0
Pneumonia	0	0	1 (3.7)
Heart failure	0	0	1 (3.7)

^a^Listed in this category were events that occurred in at least 5% of the patients in either group.

## Discussion

Our present study, as a bridging study, met its predefined success criterion (difference of VAS decreases greater than 4.13 mm, retained at least 50% of the efficacy of the key clinical trial in Japan), in line with requirements for the bridging study from the Chinese regulator. To the best of our knowledge, this is the first report on the safety and efficacy of nalfurafine in Chinese subjects. The strength of our study also included the prospective study design and hemodialysis patients from multiple centers. Thus, we could validly concluded that the efficacy results of this bridging trial were consistent with the results of the Japanese phase III trial, in accordance with the ICH guidelines [33,34]. The differences of VAS change, the study’s primary endpoint, between both nalfurafine groups and placebo group were greater than 4.13 mm, and a significant response was shown in the 5 μg nalfurafine group, whereas not in the 2.5 μg group. The Xie-kawashima itch scale was also used to measure improvement in itch severity. The treatment of nalfurafine seemed to alleviate the intensities of daytime and nighttime itching with a slightly dose-dependent manner, though there was no significant difference. The systematic review by Hercz et al. [15] found that the kappa opioid agonists, including nalfurafine, significantly reduced itch severity (6 studies, 661 participants: 10.5 mm reduction, 95% CI 1.40 to 0.71 lower in VAS compared to placebo) in the different populations with CKD-aP. The magnitude of the reduction in our study was similar to this result, indicating that the population responded in a similar way to other populations.

Although the design of our study was similar to the previous study conducted in Japan [19], it should be noted that differences exist between the two studies. The main reason of statistical results of our study differed from the Japanese study might be the different sample size: 141 subjects in our study, while 337 subjects in the Japanese one. The statistical difference of low-dose group might not come to the surface due to the small sample. Another reason was that the scoring methods of two criterias measuring the itch severity in our study were somewhat subjective, leading to statistical discrepancy, which suggested that the therapy of pruritus was susceptible to a placebo effect. Specifically, our study was a bridging study designed to show consistency in efficacy with the Japanese one, rather than to go for between-group statistical comparisons. The groups treated with nalfurafine were included to enable the verification of our results once the question ‘whether the studied population responded to nalfurafine of proved efficacy?’ can be answered by the observation of the results of the treatment groups.

A bridging study can be carried out to ‘build a bridge’ with foreign clinical data. The regulatory issues required by the Chinese National Medical Products Administration and long clinical testing and approval time for drugs to be marketed in China continue to be stumbling blocks for foreign drugs in the Chinese market. The ICH E5 guidelines [24] were introduced in 1998 and remained unchanged since that time. Adoption of the guidelines cleared the way for the clinical trial data generated in one region to be used in another for drug regulatory approval purposes, a practice termed ‘bridging’. Besides, genetic variability related to ethnicity might alter the pharmacokinetics and pharmacodynamics of drugs, resulting in difference in response to drug therapy. In the recent years, bridging studies have been required as a part of the clinical data package needed for approval of foreign drugs in the Chinese market. This strategy according to the ICH guidelines on regional and ethnic factors in the acceptance foreign clinical data allowed investigators to use bridging studies to extrapolate data from large foreign studies to smaller domestic trials [23,24,34]. As discussed above, our bridging study, using the same protocol and including less than half of sample size of the previous study conducted in Japan, achieved the predefined success criterion and confirmed the clinical benefit of the approved dose regimen (nalfurafine) for the Chinese population, without duplicating research efforts.

The pathogenesis of CKD-aP remained to be fully elucidated, thus developing effective therapeutic agents was quite tough. The current treatment options for intractable pruritus in patients with hemodialysis, which include topical treatment, antihistamines and antiallergic agents, phototherapy, specifically selected dialysis membranes, or performing hemodiafiltration, are not sufficiently effective [7,11–15]. Although the gabapentinoids, not licensed for the treatment of itch, can be considered as an effective treatment for CKD-aP [15], the adverse risks of somnolence, dizziness, and fatigue must be informed and monitored carefully [17]. One of the overarching hypothesis implicating an imbalance of opioid system had been proposed, and it emphasized that μ-opioid receptor activation and κ-opioid receptor blockade leading to pruritogenic nerve signaling and pruritogenic cytokines release [11,18,19]. It was supported by the observation that the ratio of the κ-opioid receptor agonist (dynorphin-A) to the μ-opioid receptor agonist (β-endorphin) was decreased in CKD-aP patients compared with healthy controls [35]. Hence, it led to the development of nalfurafine, an oral highly selective κ-opioid receptor agonist that has been approved in Japan for the treatment of moderate to severe CKD-aP for more than ten years.

In clinical studies, nalfurafine has been demonstrated to present a prominent efficacy for intractable pruritus in chronic liver failure [36] and uremic patients [19,22], by a novel mechanism differing from conventional drugs [35,37,38]. The κ-opioid receptors in the central nervous system (CNS) and epidermis, were considered to play an important role in the pathogenesis of severe pruritus, with a series of data obtained in animal and human experiments [39–43]. It could be concluded that nalfurafine targeted on the κ-opioid receptors in the skin, as well as in the CNS. As with all opioid treatments, the possibility of abuse and dependence was a major problem. There was no indication of developing abuse liability, physical dependence, or withdrawal effects after nalfurafine therapy in the one year long-term study [21]. Recently difelikefalin, a peripherally restricted selective κ-opioid receptor agonist for intravenous use, had a significant antipruritic effect through activation of κ-opioid receptors on peripheral neurons and interaction with k-receptors on immune cells decreasing the release of proinflammatory agents. The KALM-1 trial, a randomized, doubleblind, placebo-controlled phase 3 trial, showed that difelikefalin was effective at reducing itch intensity and improving itch-related quality of life in hemodialysis patients with moderate-to-severe CKD-aP [44]. And it led to the approval of intravenous difelikefalin in the USA for the treatment of CKD-aP [45]. The efficacy of difelikefalin was also verified in the Japanese hemodialysis patients [46]. Compared to difelikefalin, nalfurafine is more convenient with oral route for hemodialysis patients.

The safety profile of nalfurafine observed in the present study remained similar to that outlined in the studies carried out in the CKD-aP population of Japan [19,26]. Most ADRs were expected, and might be mediated by the central nervous system (insomnia and irritability) and the gastrointestinal system (nausea). The most frequent ADR in this study was insomnia, in line with the previous results [19,22,26]. Insomnia did not led to any patient dropping out in our study, while it was responsible for four subjects withdrawal from the Japanese trial [19], so particular attention should be paid to this ADR. In the present study, transient prolactin elevation was the second common event and found in 12 of 57 patients in the 5 μg group, 5 of 57 in the 2.5 μg group and 2 of 27 in the placebo group, although gynecomastia and galactorrhoea were not reported. One explanation may be that nalfurafine seems to induce endocrine changes through the common effects of opioid receptor in the CNS and cause endocrine disorders mean while [22,47]. Therefore, prolactin should be tested as appropriate during administration. SAEs occurred rarely and had nothing to do with the study drug, and individual SAEs tended to occur singularly. Since all of ADRs were transient and resolvable, this suggested that nalfurafine was a safe agent.

This trial had certain limitations. The trial enrolled patients in whose gender were male predominance in each group. Although there were total of 141 cases enrolled in our study and the sample size was considered to be effective to meet the predefined success criteria of our bridging trial, the sample size was smaller than the Japanese trial of more than 300 cases. The safety and efficacy of nalfurafine were assessed for 22 days in this trial that was similar with the Japanese trial. Assessments over longer durations including Chinese patients will help support the generalizability of this treatment.

In summary, the results of this prospective randomized, placebo-controlled multicenter bridging study did show efficacy of nalfurafine administration for treatment-resistant CKD-aP in Chinese population undergoing hemodialysis based on predefined criterion. The safety profile of nalfurafine was consistent with that observed in previous studies, and no new safety issues had been proposed. Nalfurafine was found to be an effective and safe treatment option for Chinese hemodialysis patients with pruritus in this study.

## Supplementary Material

Supplemental Material
