# Supplementary material for: Randomized controlled trial of nalfurafine for refractory pruritus in hemodialysis patients
Source: Ren Fail. 2023 Mar 1;45(1):2175590. doi: 10.1080/0886022X.2023.2175590 (PMC9980412; doi:10.1080/0886022X.2023.2175590)
Supplement: Supplemental Material [file IRNF_A_2175590_SM7831.pdf]

Table S1. Inclusion and Exclusion Criteria

Inclusion Criteria	Exclusion Criteria
(1) Chronic renal failure patients who had been on stable dialysis for 3 months or more, received regular hemodialysis 3 times a week, and expect no significant treatment changes or dramatic changes in their condition during the clinical trial;<br>(2) One year prior to signing the informed consent, patients who were treated with the following drugs A and/or B:<br>(A) Systematic treatment (oral, injection, etc.) of "prescribed drugs for pruritus" (including antihistamines or antiallergic drugs) for more than 2 weeks consecutively;<br>(B) Use of " prescribed drugs for pruritus" (ointments, etc.) or topical treatment of moisturizers prescribed by physicians;<br>(3) Patients who received treatment in inclusion criteria (2) but did not respond;<br>(4) Patients was older than 18 years old (including 18 years old), regardless of gender;<br>(5) During the observation period before administration (D8-14), the number of days for which the VAS values were measured at the time of getting up and going to bed should not be less than 5 days, and the average value of the larger VAS values should not be less than 50 mm;<br>(6) During the observation period before administration (D8-14), The number of days in which the larger VAS value measured in the morning and evening measurements (if there was a missing time in getting up or going to bed, the value which has been measured was selected) is not less than 20 mm, was not less than 5 days;<br>(7) During the observation period before administration (D8-14), the number of days in which the pruritus severity of Hsie-Kawashima's was evaluated at the time of getting up and at the time of going to bed was not less than 5 days, in which the maximum of the pruritus score was not less than 3 (moderate) in the morning and evening measurements was more than half.	(1) Patients with malignant tumor;<br>(2) Patients with mental illness or mental retardation who cannot correctly understand the VAS score and describe their feelings;<br>(3) Patients with alanine aminotransferase (ALT), aspartate aminotransferase (AST) or glutamine transferase (GGT) or total bilirubin higher than twice the upper limit of normal value (ULN) at the screening stage;<br>(4) Patients currently suffering from atopic dermatitis or chronic urticaria;<br>(5) Patients allergic to opioids;<br>(6) Patients with drug or alcohol dependence;<br>(7) Patients who had received light therapy for pruritus within 1 month prior to signing the informed consent;<br>(8) Patients who have participated in a previous clinical study of Nalfurafine Hydrochloride and have taken Nalfurafine Hydrochloride, or who have participated in this clinical study and have been officially enrolled;<br>(9) Participated in other clinical studies (including research drugs and medical devices) within 1 month before signing the informed consent;<br>(10) Pregnant women, lactating women, women who have a positive pregnancy test or who do not agree to use contraception method during the study period;<br>(11) Patients who could not have their VAS score recorded by themselves for any reason;<br>(12) Patients’ comorbidities or previous medical history would affect the evaluation of the study, as determined by the investigator;<br>(13) HIV positive;<br>(14) Other conditions judged by the investigator that were unsuitable for participation in this clinical study.

Information Classification: General

Table S2. Research design and flow chart

Screen	Pre-observation		Treatment		Post-observation
**7 day**	**First 7 day**	**Latter 7 day**	**First 7 day**	**Latter 7 day**	**8 day**
			Plus nalfurafine 5μg, 2.5μg or placebo		
	Baseline treatment without change				

Figure S1 Visual analogue scale (VAS)

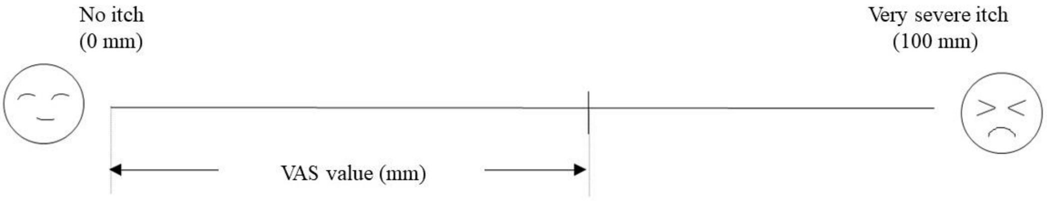

The patients were asked to mark the VAS value to record the worst itch experienced during the previous 12 h twice a day (once in the morning and once in the evening).

Randomization Schemes:

The randomization schemes are presented below:

Screening numbering scheme: The screening number consists of S+2 digits center number + 3 digits of signed informed consent sequence number (Arabic numerals), which is assigned according to the time order, starting from 001. For example, the screening number of the first subject who signed the informed consent form in the 10th center is S10001, and so on.

Subjects who meet the screening period requirements to enter the pre-observation period are pre-registered. And the pre-registration number consists of Y+2 digits center number + 3 digits of registration acceptance sequence number (Arabic numerals), according to the registration acceptance order, starting from 001 in ascending order. For example, the pre-registration number of the first pre-registered subject of the 10th center is Y10001, and so on.

Randomization was performed by persons unrelated to this study, using SAS software (version 9.4 or later) to generate a randomization table using the differential randomization method, and randomized subjects to 5μg nalfurafine group, 2.5μg nalfurafine group, and placebo group in a ratio of 2:2:1. If the selected cases meet the inclusion criteria during the official enrollment period, the investigators assign a corresponding random number using the Interactive Web Response System (IWRS) in the order of enrollment, and issue the drug according to the corresponding drug number, which will remain unchanged throughout the study. Random numbers are in the form 001, 002, 003, 004, and so on.

Information Classification: General

In order to avoid the subjective factors of the investigator or subject affecting the efficacy evaluation, a special person should be assigned to manage, distribute and recycle the drug.

Table S3. The benchmark descriptors for each level of Shiratori's severity score

daytime		nighttime	
4	unbearable itch causes scratching and scratching only aggravates the itch sensation and interferes with work or study	4	almost deprived of sleep, the patient keeps scratching ceaselessly, which only aggravates the itch sensation
3	significant scratching even in public or irritating itch causes ceaseless scratching	3	the itch disrupts sleep, and although the patient can go back into sleep after scratching, the patient keeps scratching unconsciously
2	itch that can be subdued by occasional reaching out and light scratching	2	occasional itch that can be subdued by scratching and does not wake the patient
1	occasional restless sensations that do not necessarily induce scratching behavior	1	very little itch at bedtime that does not induce conscious scratching behavior and never disturbs sound sleep
0	no or almost no itch	0	no or almost no itch

Information Classification: General
